# Short-term insulin intensive therapy decreases MCP-1 and NF-κB expression of peripheral blood monocyte and the serum MCP-1 concentration in newlydiagnosed type 2 diabetics

**DOI:** 10.20945/2359-3997000000029

**Published:** 2018-03-23

**Authors:** Yang Lin, Shandong Ye, Yuanyuan He, Sumei Li, Yan Chen, Zhimin Zhai

**Affiliations:** 1 Shandong University Shandong University School of Medicine Jinan Shandong China School of Medicine, Shandong University, Jinan, Shandong 250100, China; 2 Anhui Provincial Hospital Department of Pediatrics Hefei Anhui China Department of Pediatrics, Anhui Provincial Hospital, Hefei, Anhui 230001, China; 3 Anhui Provincial Hospital Department of Endocrinology Hefei Anhui China Department of Endocrinology, Anhui Provincial Hospital, Hefei, Anhui 230001, China; 4 Anhui Provincial Hospital Endocrinological Laboratory Hefei Anhui China Endocrinological Laboratory, Anhui Provincial Hospital, Hefei, Anhui 230001, China; 5 Anhui Provincial Hospital Department of Central lab Hefei Anhui China Department of Central lab, Anhui Provincial Hospital, Hefei, Anhui 230001, China

**Keywords:** Type 2 diabetes, insulin intensive therapy, MCP-1, NF-κB

## Abstract

**Objective:**

To observe the effect of short-term insulin intensive treatment on the monocyte chemoattractant protein-1 (MCP-1) as well as on the nuclear factor-kappa B (NF-κB) expression of peripheral blood monocyte. This is also in addition to observing the serum MCP-1 level in newlydiagnosed type 2 diabetic patients and probing its anti-inflammation effects.

**Subjects and methods:**

Twenty newly-diagnosed type 2 diabetic patients were treated with an insulin intensive treatment for 2 weeks. MCP-1 and NF-κB expression on the monocyte surface were measured with flow cytometry, the serum MCP-1 level was measured by enzyme linked immunosorbent assay (ELISA) during pretreatment and post-treatment.

**Results:**

After 2 weeks of the treatment, MCP-1 and NF-κB protein expression of peripheral blood monocyte and serum MCP-1 levels decreased significantly compared with those of pre-treatment, which were (0.50 ± 0.18)% vs (0.89 ± 0.26)% (12.22 ± 2.80)% vs (15.53 ± 2.49)% and (44.53 ± 3.97) pg/mL vs (49.53 ± 3.47) pg/mL, respectively (P < 0.01). The MCP-1 expression on monocyte surface had a significant positive relationship with serum MCP-1 levels (r = 0.47, P < 0.01).

**Conclusions:**

Short-term insulin intensive therapy plays a role in alleviating the increased inflammation reaction in type 2 diabetics.

## INTRODUCTION

The world has seen a rapid increase in the incidence of diabetes generally, and type 2 diabetes mellitus (T2DM) has become the most common metabolic disease globally (1). Experimental data have shown that hyperglycemia may induce a chronic inflammatory state in the vessel wall, thus accelerating the development of diabetic chronic complications (2). T2DM and obesity are insulin-resistant (IR) disorders characterized by chronic inflammation (3).

NF-κB is a family of transcription factors that controls the production of pro-inflammatory proteins. The NF-κB pathway unites the inflammatory and metabolic responses, and, as a well-studied mediator of inflammation, represents an entry point for an improved understanding of metabolic diseases. MCP-1 is a member of chemotactic cytokines (CC) sub-family chemokines, which plays a pivotal role in the development of inflammatory reactions. MCP-1 accelerates the development and progress of diabetic nephropathy (DN) and macrovascular complications through inducing inflammatory cells from the circulation to artery endothelium and kidney. It also contributes to the proliferation of arterial smooth muscle cells, which constitute the key cellular components of atherosclerotic plaques (4).

Insulin has been shown to suppress inflammatory changes through the down-regulation of proinflammatory cytokines, reactive oxygen species (ROS) (5) and acute phase proteins (6), and the up-regulation of anti-inflammatory cytokines (7). In this report, we aim to demonstrate an anti-inflammatory effect of short insulin intensive therapy through comparing changes in MCP-1 expression on the monocyte surface, NF-κB expression in the monocyte, and serum MCP-1 level in newly-diagnosed T2DM patients pre-short-term insulin intensive treatment with those of post-treatment.

## SUBJECTS AND METHODS

The subjects are 20 newly-diagnosed T2DM patients (HbA1c 9.02 ± 0.17%, women, n = 6; men, n = 14; mean age, 51.25 ± 5.71 years) admitted to department of endocrinology, Anhui provincial hospital, which is consistent with the diabetes diagnostic criteria of the World Health Organization (WHO) in 1999. To reduce the possibility of confounding, patients were selected according to the following exclusion criteria: pregnancy, concomitant diseases, smoking, and alcohol intake of more than 10 g/d. Patients with allergic diathesis regarding both type 1 e type 2 immune responses and/or reporting respiratory, gastrointestinal, or genitourinary tracts infections during the last three months were also screened out. The acute complications and severe chronic complications of diabetes have not appeared in type 2 diabetics recently, and the urinary albumin relative to the urinary creatinine was below 300 mg/gCr. None of the patients were receiving statins, aldosterone receptor antagonists, angiotensin-converting enzyme inhibitors, or angiotensin receptor antagonists, antibiotics, nonsteroidal anti-inflammatory drugs, corticosteroids, or cytotoxic drugs at the time of the study. The other 20 healthy volunteers (women, n = 9; men, n = 11; mean age, 54.30 ± 7.39 years) were chosen from the Health Medical Examination Center of Anhui Provincial Hospital, serving as control subjects.

### Research design

Blood samples were obtained from all participants after a 12-hour fast to measure the blood glucose, insulin, glycosylated haemoglobin A1c (HbA1c), white blood cell count (WBC), the neutrocyte percents (N%), cholesterol (TC), triglyceride (TG), low density lipoprotein cholesterol (LDL-C), MCP-1 expression on the monocyte surface, serum MCP-1 level and NF-κB expression in monocyte. Random spot urine samples were collected for the examination of urinary Albumin and creatinine levels. To eliminate the effect of urine volume, we expressed the level of urinary albumin (UALB) relative to the urinary creatinine (UCr) excretion, which was abbreviated as UACR (UALB/UCr ratio). Clearance of Creatinine Rate (Ccr) was calculated to stand for the glomerular filtration rate (GFR), using the formula raised by Cockcroft and Gault in 1976 (8). The patients in the observation group then received insulin intensive treatment consisting of regular pre-meal and bedtime neutral protamine hagedorn (NPH) insulin injections for two weeks. The indexes mentioned above were measured again at the study's conclusion. All laboratory tests were performed in the Biochemical Laboratory and Endocrinology Laboratory of Anhui Provincial Hospital.

The 20 newly-diagnosed T2DM patients, with an average HbA1c (9.02%), indicated that they had poor blood-glucose control, so the insulin treatment was necessary. Fasting blood glucose (FBG) and postprandial 2h blood glucose (P2hBG) were measured daily, and daily insulin doses (in units) were approximately 1 unit per kilogram; the dosage of insulin was adjusted according to blood glucose levels, FBG were controlled among 3.9~8.3 mmol/L while P2hBG were controlled < 10 mmol/L, and 3-5 days were limited to reach the determined blood glucose levels.

## METHODS

### Measurement of Serum MCP-1 Level (by ELISA)

Fasting serum samples were collected to measure serum MCP-1 level. For purposes of analysis, samples were thawed and vortexed vigorously, and MCP-1 level (pg/mL) was measured in duplicate according to the manufacturer's instructions with a commercially available ELISA (Biosource Inc).

### Measurement of MCP-1 expression on the monocyte surface (by flow cytometry)

Five mL peripheral vein blood were drawn with the heparin supplemented tube. The measurement tube was supplemented with mouse monoclonal antibody to the human CD14 antigen stained with PE-Cy5 (Caltag Laboratories) 2.5 ul and mouse monoclonal antibody to the human MCP-1 antigen stained with PE (eBioscience) 1.25 ul. Mouse monoclonal antibody was added in the control tube to the human CD14 antigen stained with PE-Cy5 2.5 ul and Armenian hamster IgG isotype control stained with PE (eBioscience) 1.25 ul, then both the measurement tube and the control tube were injected with 100 ul blood followed by shaking in order to misce bene. Subsequently, both tubes were protected from light for 20 min at room temperature, and then each tube was added with hemolytic agent 2 mL. 10 min later, the two tubes were centrifugated at 1500 r/min for 5 min, abandoned the supernatant and supplemented with 2 mL PBS, repeated 1500 r/min centrifugalization for 5 min. Each tube was then washed three times with PBS, abandoned the supernatant, and then both the two tubes were supplemented with normal sodium to 500 ul, shaken to misce bene before measured by flow cytometry.

### Measurement of NF-κB expression in the monocyte (by flow cytometry)

One milligram peripheral vein blood were drawn with heparin supplemented tube. The measurement tube and the control tube were supplemented with mouse monoclonal antibody to the human CD14 antigen stained with PE-Cy5 (Caltag Laboratories) 2.5 ul, then were added with 100 ul blood followed by shaking in order to misce bene. Subsequently, both tubes were protected from light for 20 min at room temperature, and then the tubes were added with 100 ul Fixation Medium A to fix. The tubes were then protected from light for 15 min at room temperature, then supplemented with 2 mL PBS, followed by 1500 r/min centrifugalization for 5 min, abandoned the supernatant and supplemented with 100 ul permeabilization medium B to rupture the membrane of monocyte. The measurement tube was added with mouse monoclonal antibody to the human NF-κB P65 antigen that stained with PE (eBioscience) 5 ul while the control tube had Armenian hamster IgG isotype control stained with PE added (eBioscience) 5 ul, followed by shaking in order to misce bene. Following this, both tubes were protected from light for 20 min at room temperature, supplemented with 2 mL PBS, repeated 1500 r/min centrifugalization for 5 min, and abandoned the supernatant. After this, both tubes were supplemented with normal sodium to 500 ul, shaked to misce bene before measured by flow cytometry.

### Statistical analysis

Data were presented as means ± SD and analyzed using the Statistical Package for the Social Sciences 13.0. After testing data for normality, we used Student's paired *t* test to compare values before and after two weeks of insulin intensive treatment. The relationship between MCP-1 expression on monocyte surface and serum MCP-1 level, the relationships between MCP-1, NF-κB, and the change of FBG and P2hBG were performed by linear correlation. In all cases, a *P* value ≤ 0.05 was considered statistically significant.

## RESULTS

### The comparisons of data between diabetic patients and control group

**Figure 1:**
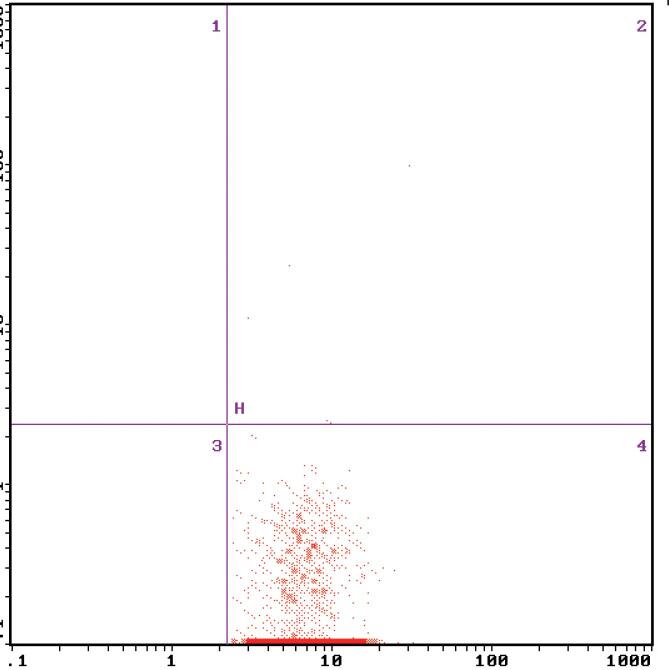
The MCP-1 expression on monocyte surface measured by flow cytometry in normal control group.

**Figure 2:**
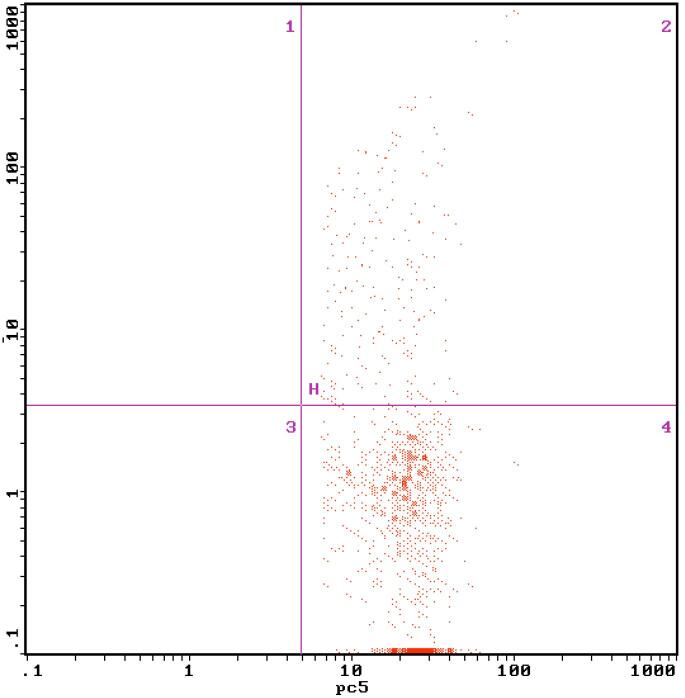
The MCP-1 expression on monocyte surface measured by flow cytometry before the treatment

The FBG, P2hBG, HbA1C, TC, TG, LDL-C, UACR, the expression of MCP-1 on monocyte and NF-κB in monocyte and serum MCP-1 level in diabetic patients were higher significantly than those in the control group, which were (11.18 ± 1.73) mmol/L vs (4.52 ± 0.15) mmol/L (17.92 ± 2.08) mmol/L vs (5.26 ± 0.34) mmol/L (9.02 ± 0.17)% vs (5.37 ± 0.08)% (5.45 ± 0.65) mmol/L vs (4.51 ± 0.53) mmol/L (2.02 ± 0.35) mmol/L vs (1.40 ± 0.30) mmol/L (3.85 ± 0.59) mmol/L vs (2.32 ± 0.69) mmol/L (71.98 ± 31.62) mg/gCr vs (12.88 ± 1.35) mg/gCr (0.89 ± 0.26)% vs (0.28 ± 0.09)% (Figures 1 and 2), (15.53 ± 2.49)% vs (3.67 ± 1.68)% (Figures 4 and 5) (49.53 ± 3.47) pg/mL vs (39.14 ± 7.43) pg/mL, respectively, P < 0.05. But there were no significant differences in age, BMI, GFR, SBP and DBP between the diabetic patients and the control group, which were (51.25 ± 5.71)y vs (54.30 ± 7.39)y, (23.26 ± 0.40) kg/m^2^ vs (22.15 ± 0.62)kg/m^2^, (99.35 ± 10.30) mL/min vs (105.10 ± 8.74) mL/min, (128.63 ± 2.41) mmHg vs (125.28 ± 5.31) mmHg, (75.21 ± 2.12) mmHg vs (70.31±2.48) mmHg, respectively, P > 0.05 (Table 1).

**Table 1:** The comparisons of paremeters between diabetic patients and control group

	NC	DM
AGE (y)	54.30 ± 7.39	51.25 ± 5.71
BMI (kg/m^2^)	22.15 ± 0.62	23.26 ± 0.40
SBP (mmHg)	125.28 ± 5.31	128.63 ± 2.41
DBP (mmHg)	70.31 ± 2.48	75.21 ± 2.12
TC (mmol/L)	4.51 ± 0.53	5.45 ± 0.65*
TG (mmol/L)	1.40 ± 0.30	2.02 ± 0.35*
LDL-C (mmol/L)	2.32 ± 0.69	3.85 ± 0.59*
GFR (mL/min)	105.10 ± 8.74	99.35 ± 10.30
UACR (mg/gCr)	12.88 ± 1.35	71.98 ± 31.62*
FBG (mmol/L)	4.52 ± 0.15	11.18 ± 1.73*
P2hBG (mmol/L)	5.26 ± 0.34	17.92 ± 2.08*
HbA1c (%)	5.37 ± 0.08	9.02 ± 0.17*
serum MCP-1 (pg/mL)	39.14 ± 7.43	49.53 ± 3.47*
MCP-1 expression (%)	0.28 ± 0.09	0.89 ± 0.26*
NF-κB expression in monocyte (%)	3.67 ± 1.68	15.53 ± 2.49*

vs normal control group,

* P < 0.05.

**Figure 3:**
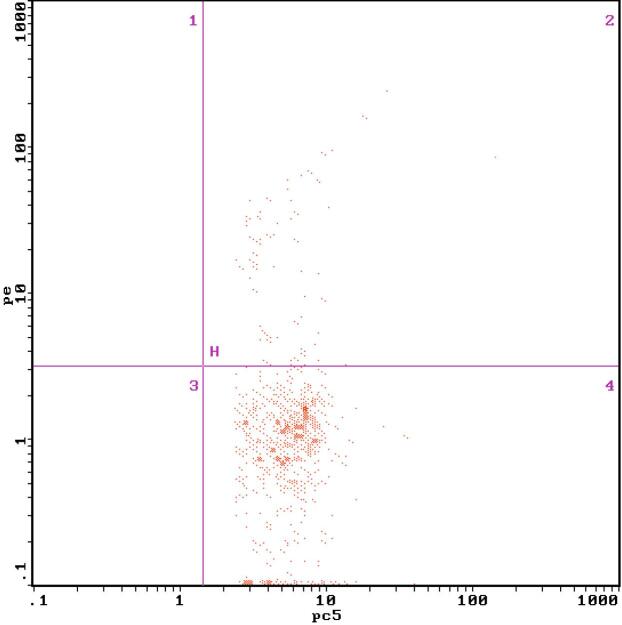
The MCP-1 expression on monocyte surface measured by flow cytomery after the treatment.

The changes of the MCP-1 and NF-κB expression before and after insulin intensive therapy in diabetic group are as follows:

After 2 weeks of the insulin intensive treatment, the MCP-1 expression on monocyte surface and serum MCP-1 level decreased significantly compared with those of pre-treatment, which were (0.50 ± 0.18)% vs (0.89 ± 0.26)%, (44.53 ± 3.97) pg/mL vs (49.53 ± 3.47) pg/mL, respectively, (P < 0.01) (Table 2, Figures 2 and 3); NF-κB expression in monocyte also decreased significantly compared that of pre-treatment, which were (12.22 ± 2.80)% vs (15.53 ± 2.49)% (P < 0.01) (Table 2, Figures 5 and 6).

**Table 2:** The changes of indexes in diabetic group before and after insulin intensive therapy

	N	pre-treatment	post-treatment	t	p
FBG (mmol/L)	20	11.18 ± 1.73	6.37 ± 1.07	11.77	< 0.01
P2hBG (mmol/L)	20	17.92 ± 2.08	8.27 ± 2.12	19.14	< 0.01
Homa-IR(log)	20	0.78 ± 0.03	0.71 ± 0.04	6.43	< 0.01
WBC × 109/L	20	7.39 ± 1.56	5.52 ± 1.12	5.54	< 0.01
N%	20	64.61 ± 5.45	56.23 ± 4.71	5.72	< 0.01
BMI (kg/m^2^)	20	23.26 ± 0.40	22.43 ± 0.52	1.856	> 0.05
TC (mmol/L)	20	5.45 ± 0.65	5.32 ± 0.51	2.77	< 0.05
TG (mmol/L)	20	2.02 ± 0.35	1.82 ± 0.31	8.91	< 0.01
LDL-C (mmol/L)	20	3.85 ± 0.59	3.55 ± 0.53	6.88	< 0.01
GFR (mL/min)	20	99.35 ± 10.30	101.20 ± 8.84	1.78	> 0.05
UACR (mg/gCr)	20	71.98 ± 31.62	53.52 ± 24.99	6.50	< 0.01
serum MCP-1 level (pg/mL)	20	49.53 ± 3.47	44.53 ± 3.97	4.21	< 0.01
MCP-1 expression (%)	20	0.89 ± 0.26	0.50 ± 0.18	8.48	< 0.01
NF-κB expression in monocyte (%)	20	15.53 ± 2.49	12.22 ± 2.80	3.96	< 0.01

**Figure 4:**
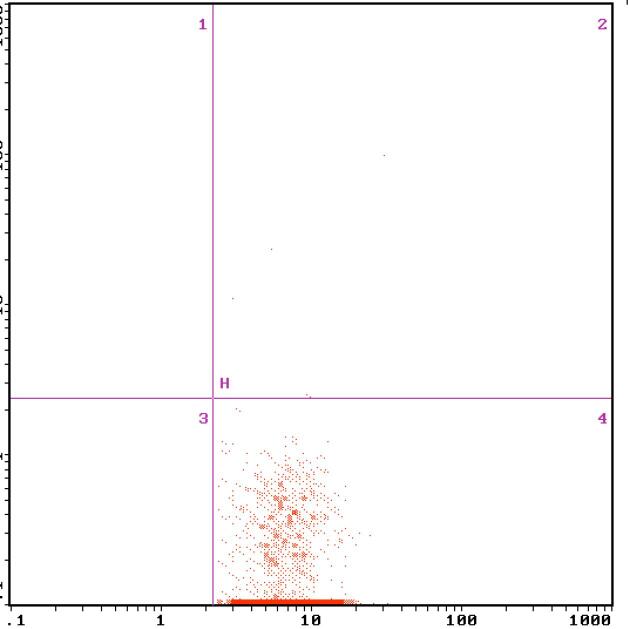
The expression of monocyte NF-κB measured by flow cytometry in normal control group.

**Figure 5:**
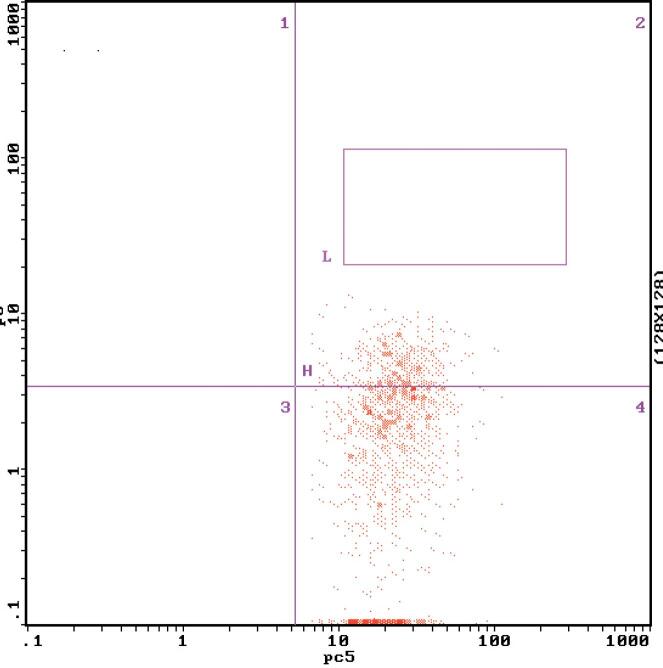
The expression of monocyte NF-κB measured by flow cytometry before the treatment.

**Figure 6:**
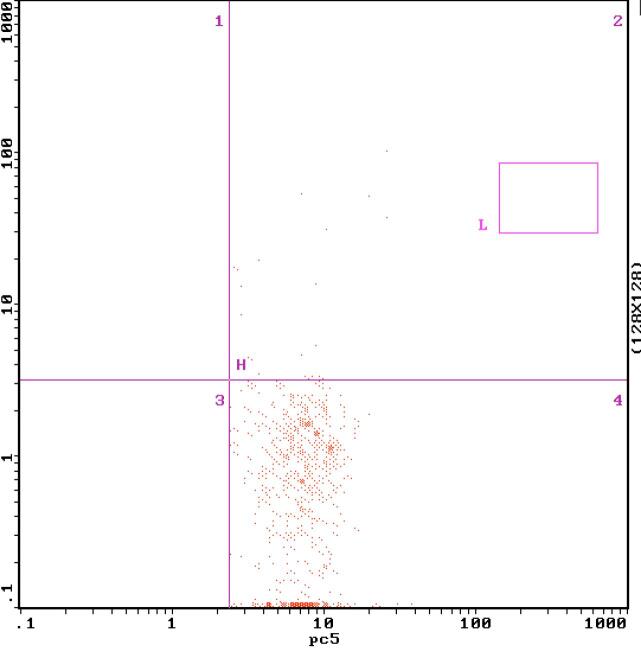
The expression of monocyte NF-κB measured by flow cytometry after the treatment

The changes of FBG, P2hBG, Homa-IR (log), WBC and N% before and after insulin intensive therapy in diabetic group are as follows:

The FBG, P2hBG, Homa-IR(log), WBC and N% all significantly decreased after two weeks of insulin intensive treatment compared with those of pretreatment, which were (6.37 ± 1.07) mmol/L vs (11.18 ± 1.73) mmol/L, (8.27 ± 2.12) mmol/L vs (17.92 ± 2.08) mmol/L, (0.71 ± 0.04) vs (0.78 ± 0.03), (5.52 ± 1.12) × 10^9^/L vs (7.39 ± 1.56) × 10^9^/L, (56.23 ± 4.71)% vs (64.61 ± 5.45)% (Table 2).

### Relationship analysis

The MCP-1 expression on monocyte surface had a significant and positive relationship with serum MCP-1 level (*r* = 0.47, *P* < 0.01).

The expression of NF-κB in monocyte had a positive relationship with the MCP-1 expression on monocyte surface (*r* = 0.51, *P* < 0.01) and serum MCP-1 level (*r* = 0.49, *P* < 0.01).

The changes of FBG had no significant relationship with the changes of MCP-1 expression on monocyte surface (*r* = 0.31 *P* > 0.05), serum MCP-1 (*r* = 0.33, *P* > 0.05) and expression of NF-κB in monocyte (*r* = 0.28, *P* > 0.05).

The changes of P2hBG had no significant relationship with the changes of MCP-1 expression on monocyte surface (*r* = 0.32, *P* > 0.05), serum MCP-1 (*r* = 0.28, *P* > 0.05) and expression of NF-κB in monocyte (*r* = 0.31, *P* > 0.05).

### The incidence of hypoglycemia

Hypoglycemia was recorded as blood glucose < 3.9 mmol/L with symptoms such as hunger, shakiness, nervousness, sweating, or dizziness. Twenty-six percent of patients were recorded to have had a hypoglycemic attack at least once in the T2DM patients. No hypoglycemia occurred in the control group.

## DISCUSSION

T2DM is characterized by two major defects: a dysregulation of pancreatic hormone secretion (quantitative and qualitative – early phase, pulsatility – decrease of insulin secretion, increase in glucagon secretion), and a decrease of insulin action on target tissues (IR) (9). Now, T2DM is also recognized as a disease of chronic inflammation, which may play an important role in the development of IR, impairment of islet function, and diabetic chronic complications (10). Hyperglycemia, a main metabolic disorder in T2DM patients, activates the body's inflammatory defense mechanism, causing the waterfall release of numerous inflammatory mediators and cytokines, and eventually leading to organ damage (11). The dyslipidemia of T2DM also results in an increase in circulation concentrations of inflammatory cytokines (12). Previous studies have shown that many inflammatory factors, such as NF-κB, MCP-1, TNF-α, C reactive protein (CRP), IL-6, etc., not only take part in the dys-regulation of pancreatic hormone secretion and IR, but also in the development of the chronic complications of diabetes, such as its microvascular and macrovascular complications (12). In this study, the result also showed that the expressions of MCP-1 on monocyte and NF-κB in monocyte and serum MCP-1 level increased significantly in newly-diagnosed diabetic patients, which further indicates that increased chronic inflammation is present in T2DM patients.

**Figure 7:**
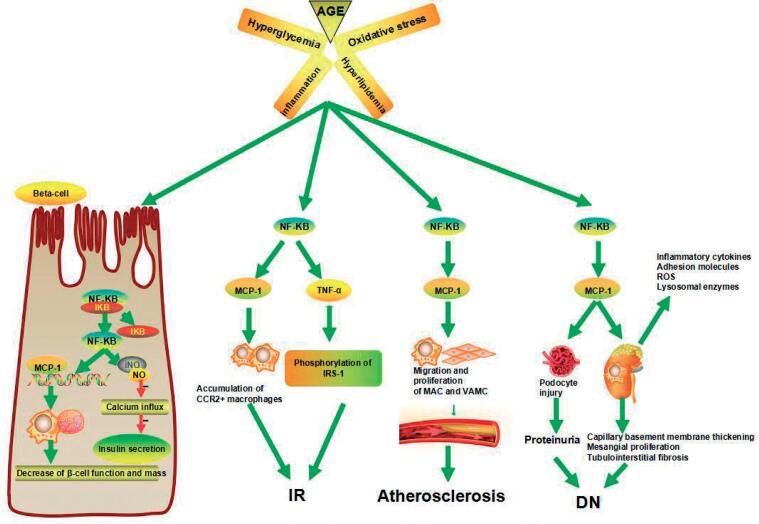
The pathophysiology of NF-κB and MCP-1 in T2DM.

Data accumulated in recent years suggest that activated NF-κB signaling pathway may be involved in the pathogenesis of IR, stress response, and inflammation by controlling gene network expression (13–15). NF-κB is present in the cytoplasm complexed to its inhibitory protein known as inhibitory κB (Iκ-B). Following activation, the phosphorylation of IκB molecules promotes their degradation and the release of NF-κB, which then translocates to the nucleus to promote the transcription of target genes (16). The NF-κB signaling pathway controls the expression of genes including inflammatory cytokines, chemokines, and adhesion molecules (17), such as MCP-1, IL-6, TNF-α, etc., which in turn initiate local inflammation and leukocyte accumulation (18).

MCP-1 is expressed by a variety of cell types, including monocyte, macrophage, endothelial cell (EC), vascular smooth muscle cell (VSMC), podocyte, adipocyte and fibroblast, etc. The expression of MCP-1 in all kinds of cells listed above is low under normal physiological conditions, once given the stimulations such as increased blood glucose, advanced glycation end products (AGEs), hyper-lipemia, angiotensin II (Ang II), oxidative stress, interleukin-1 (IL-1), TNF-α, et al., the expression of MCP-1 is obviously up-regulated (19,20). Increased serum MCP-1 level in humans correlates with markers of metabolic disorder including obesity, IR, and T2DM (21–23). MCP-1 accelerates the onset of atherosclerosis, as it attracts monocytes to the inflammatory sites of vascular subendothelial space, initiating the migration of monocytes into the arterial wall to form excessive macrophage-derived foam cells (24). It also participates in the early renal injury through activation of lysosomal enzymes and proteolytic enzymes in mononuclear macrophages and overproduction of ROS (25). Chow and cols. (26) reported that the MCP-1 genetic knockout mouse displayed amelioration of diabetic albuminuria, reduction in renal fibrosis, preservation of kidney clearance function, and decreased accumulation of macrophages.

Increasing evidence shows that short-term insulin intensive therapy can alleviate glucose toxicity, lipotoxicity and IR, and improve islet β cell function in newly diagnosed type 2 diabetics, but the study about its effect in inhibiting the MCP-1 and NF-κB expression in diabetic patients in vivo has not been reported. Two large, prospective, randomized controlled studies showed that intensive insulin therapy in hyperglycemic patients in a surgical intensive care unit (ICU) resulted in a 50% reduction in mortality, along with several other benefits, including a reduction in the incidence of renal failure, septicemia and ICU neuropathy. Insulin infusion also lowered circulating levels of MCP-1, intracellular adhesion molecule-1 (ICAM-1) and E-selectin in patients with prolonged critical illness (27,28). Aljada and cols. (29) reported that insulin could inhibit NF-κB and MCP-1 expression in human aortic endothelial cells. Treatment of Akita mice with insulin implants for 20 weeks normalized hyperglycemia and decreased urinary albumin excretion (30). This study showed that two weeks of intensive insulin therapy reduced MCP-1 expression on the monocyte surface, serum MCP-1 level, NF-κB expression in monocyte, peripheral blood WBC and N%, which further demonstrated the antiinflammatory effect of insulin therapy. So an earlier initiation of insulin intensive treatment may be more effective in providing long-term efficacy against the complications of T2DM.

The mechanisms underlying the anti-inflammatory effect of insulin therapy are, however, unclear, but it can be proved as follows: 1) Insulin can activate some signaling pathways, including PI3K, protein kinase B/Akt, and mitogen-activated protein kinases (MAPK) such as p38, p42/p44, and JNK (31), activation of those signaling pathway is known to limit proinflammatory gene expression (28,32–34). 2) Insulin therapy has been reported to increase the production and release of anti-inflammatory cytokines such as IL-2 and IL-4 IL-10, and anti-inflammatory signal transcription factor mRNA expression of SOCS-3 and RANTES (35). 3) Long-term hyperglycemia has been proven to activate NF-κB and upregulate the expression of MCP-1 gene, insulin intensive treatment can control hyperglycemia more quickly (36). 4) Hyperglycemia can induce intracellular oxidative stress which results in the expression of pro-inflammatory cytokines gene. Dandona and cols. (37) showed that insulin therapy could decrease the production of ROS. Recent research also confirms that insulin played a significant role in the regulation of the cellular redox balance via Nrf-2 dependent pathway (38–40). 5) Insulin therapy can attenuate the IR, which is related to the inflammatory reaction in vivo. In this trial, we also found that short-term insulin intensive therapy improved IR in newly-diagnosed T2DM patients. 6) The dyslipidemia of T2DM also results in an increase in circulation concentrations of inflammatory cytokines (41). Hayashi (42) demonstrates that intensive insulin therapy reduced serum small dense LDL particles and TG levels in type 2 diabetics. In the present study, we also found that serum TC, TG, LDL-C levels in diabetic patients were higher than those in the control group, and, furthermore, their levels decreased after two weeks of intensive insulin therapy, which indicated that insulin may exert an anti-inflammatory effect partly through its hypolipidemic effects.

In this study, we observed that the UACR was also reduced in type 2 diabetics after two weeks of treatment, which indicates that insulin intensive treatment could ameliorate micro-albuminuria, which may be partially related to the reduction of the expression of MCP-1 and NF-κB.

In summary, our primary study shows that insulin therapy can reduce the MCP-1 expression on the monocyte surface, serum MCP-1 level and NF-κB expression in monocyte, which indicates that short-term insulin intensive treatment can alleviate the increased micro-inflammatory state in T2DM patients. But the mechanisms of insulin therapy in anti-inflammation must be further evaluated. We also discovered that reduced expression of MCP-1 and NF-κB were not related to the changes of FBG and P2hBG, which requires more cases and long-term studies to research whether or not the anti-inflammatory effect of insulin therapy is independent of its hypoglycemic effects.
